# Influence of Prolonged Whole Egg Supplementation on Insulin-like Growth Factor 1 and Short-Chain Fatty Acids Product: Implications for Human Health and Gut Microbiota

**DOI:** 10.3390/nu15224804

**Published:** 2023-11-16

**Authors:** Sophida Suta, Suphawan Ophakas, Thamonwan Manosan, Orranich Honwichit, Suvimol Charoensiddhi, Apinya Surawit, Tanyaporn Pongkunakorn, Sureeporn Pumeiam, Pichanun Mongkolsucharitkul, Bonggochpass Pinsawas, Sawannee Sutheeworapong, Patcha Puangsombat, Sakda Khoomrung, Korapat Mayurasakorn

**Affiliations:** 1 Siriraj Population Health and Nutrition Research Group, Faculty of Medicine Siriraj Hospital, Mahidol University, Bangkok 10700, Thailand; sophida.sut@mahidol.edu (S.S.); phakhaun1234@gmail.com (S.O.); thamonwan.manosan@gmail.com (T.M.); apinya.sua@mahidol.edu (A.S.); tanyaporn.pon@mahidol.edu (T.P.); sureeporn.pum@mahidol.edu (S.P.); pichanun.mon@mahidol.edu (P.M.); bonggochpass.pin@mahidol.edu (B.P.); 2 Department of Food Science and Technology, Faculty of Agro-Industry, Kasetsart University, Bangkok 10900, Thailand; orranich.ho@gmail.com (O.H.); suvimol.ch@ku.th (S.C.); 3 Systems Biology and Bioinformatics Research Unit, Pilot Plant Development and Training Institute, King Mongkut’s University of Technology Thonburi, Bangkok 10140, Thailand; sawannee.sut@mail.kmutt.ac.th; 4 Metabolomics and Systems Biology, Department of Biochemistry, Faculty of Medicine Siriraj Hospital, Mahidol University, Bangkok 10700, Thailand; patcha.pou@mahidol.edu (P.P.); sakda.kho@mahidol.edu (S.K.); 5 Siriraj Metabolomics and Phenomics Center, Faculty of Medicine Siriraj Hospital, Mahidol University, Bangkok 10700, Thailand

**Keywords:** gut microbiome, metabolite, acetate, propionate, butyrate, egg consumption

## Abstract

The gut microbiota exert a profound influence on human health and metabolism, with microbial metabolites playing a pivotal role in shaping host physiology. This study investigated the impact of prolonged egg supplementation on insulin-like growth factor 1 (IGF-1) and circulating short-chain fatty acids (SCFAs). In a subset of a cluster-randomized trial, participants aged 8–14 years were randomly assigned into three groups: (1) Whole Egg (WE)—consuming 10 additional eggs per week [*n* = 24], (2) Protein Substitute (PS)—consuming yolk-free egg substitute equivalent to 10 eggs per week [*n* = 25], and (3) Control Group (C) [*n* = 26]. At week 35, IGF-1 levels in WE significantly increased (66.6 ± 27.7 ng/mL, *p* < 0.05) compared to C, with positive SCFA correlations, except acetate. Acetate was stable in WE, increasing in PS and C. Significant propionate differences occurred between WE and PS (14.8 ± 5.6 μmol/L, *p* = 0.010). WE exhibited notable changes in the relative abundance of the *Bifidobacterium* and *Prevotella* genera. Strong positive SCFA correlations were observed with *MAT-CR-H4-C10* and *Libanicoccus*, while *Roseburia, Terrisporobacter, Clostridia_UCG-014*, and *Coprococcus* showed negative correlations. In conclusion, whole egg supplementation improves growth factors that may be related to bone formation and growth; it may also promote benefits to gut microbiota but may not affect SCFAs.

## 1. Introduction

In 2022, the World Health Organization (WHO) reported that globally, 148 million (22.3%) children under the age of five were stunted, and 45 million (6.8%) were wasted. More than three-quarters of all children with severe wasting live in Southern Asia and Africa [1]. The underlying factors contributing to these conditions often stem from inadequate nutrition, including imbalances in the intake of essential macronutrients and micronutrients. Among the various dietary options, eggs emerge as a widespread source of sustenance due to their rich reservoir of high-quality protein. Furthermore, eggs are a rich source of essential micronutrients, including vitamins A, D, E, K, various minerals, and choline [2].

In our recent publication, we demonstrated that whole egg supplementation improved growth, reduced the prevalence of stunting, and enhanced nutrition biomarkers without adverse effects on blood lipoprotein levels. Intriguingly, we identified a notable increase in the abundance of specific gut microbiota. Noteworthy genera like *Bifidobacterium*, *Prevotella*, and *Lachnospira* demonstrated heightened presence [3]. However, a critical gap in knowledge remains. The impact of whole egg supplementation on insulin-like growth factor 1 (IGF-1) levels, a pivotal growth factor governing skeletal development and the promotion of bone formation [4], as well as its influence on microbiota-derived metabolites, has not been comprehensively evaluated.

Currently, a multitude of studies have reported that sufficient protein intake may contribute to skeletal health by enhancing calcium absorption and increasing circulating IGF-1 levels in the bloodstream [5,6,7,8,9]. Although the relationship between circulating IGF-1 levels and bone formation as well as skeletal development remains unclear, some studies have indicated a positive association between serum IGF-1 levels and bone mineral content among healthy children [10]. Hua et al. conducted a study showing that serum IGF-1 in children exhibited a positive correlation with growth and development. Importantly, they suggested that effective clinical interventions could significantly enhance these indicators in children to promote their growth and development [11].

In recent years, the impact of an individual’s dietary choices on changes in the gut microbiota has gained considerable attention [12]. Diet is one of the most important factors influencing the gut microbiota composition and function [13,14], which plays an important role in maintaining the host’s health and impacting nutritional status and disorders through various mechanisms [15]. Remarkably, the specific types and quantities of dietary components consumed yield a remarkable influence over the composition of the gut microbiota and consequently on the type and amount of microbiota-derived metabolites, such as short-chain fatty acid (SCFAs) and branched-chain fatty acids (BCFAs), that are produced. SCFAs, encompassing acetate, propionate, and butyrate, are aliphatic organic acids that are typically present in a 3:1:1 ratio [16,17]. These compounds result from bacterial fermentation processes that stem from indigestible carbohydrates derived from dietary fiber, including polysaccharides, oligosaccharides, and resistant starch [18]. On the other hand, BCFAs, such as isobutyrate, 2-methylbutyrate, and isovalerate, are found in lower concentrations compared to SCFAs, and their origins are exclusive to the breakdown of dietary proteins [19]. Notably, certain studies have indicated that high-protein diets can lead to elevated levels of intestinal BCFAs [20]. Additionally, Mu et al. conducted an in vivo study demonstrating that a high-protein diet can result in the reduction of bacteria responsible for producing propionate and butyrate, ultimately affecting the production of these SCFAs [21,22]. However, there exists a lack of data regarding the effects of whole egg supplementation on SCFA metabolites and their relationship with the gut microbiota in human populations. Therefore, the primary objective of this study is to investigate the influence of egg supplementation on circulating IGF-1 levels, the production of SCFAs, and the involved relationships between SCFAs and the gut microbiota within the child population.

## 2. Materials and Methods

### 2.1. Study Design

The study protocol received approval from the Institutional Review Board of Siriraj Hospital, Mahidol University (COA No. Si 322/2017), and was registered with Clinicaltrials.gov (Protocol NCT04896996) accessed on 11 October 2023. Written informed consent was obtained from the parents or legal guardians of the participating children prior to commencing the study, with their identities being kept confidential. A previous study described the participants’ flowchart and interventions [3]. Briefly, this study used a cluster randomized controlled trial with parallel design. Eligible participants, aged 8–14 years, were recruited from six rural primary schools in Thailand and randomly assigned to three groups based on weight-for-age criteria to ensure that all groups were homogeneous: (1) whole egg (WE)—consumed 10 additional whole chicken eggs per week, (2) protein substitute (PS)—consumed a yolk-free egg substitute equivalent to 10 eggs per week, and (3) control group. Participants were tracked for 35 weeks.

### 2.2. Participants and Intervention

We enlisted students from six rural primary schools, with eligibility requirements stipulating an age range of 8 to 14 years. Individuals with an egg allergy were excluded. Cluster randomization was employed, with each classroom in every school being assigned to one of the three groups. This approach aimed to minimize group confusion and maintain adherence to the groups. All six schools were encouraged to offer similar school lunch menus, when possible, to standardize caloric and nutritional content in accordance with the national school lunch program [23].

Before the intervention, all participants were instructed to maintain their usual egg consumption and dietary cholesterol intake for four weeks (washout period [week − 4]). Those in the intervention groups (WE and PS) continued their normal dietary routines. The intervention was administered individually to each classroom during regular lunch hours. The WE group received ready-to-eat commercial menus (S.W. Foodtech., Co., Ltd., Bangkok, Thailand) comprising items like hard-boiled whole eggs, scrambled eggs, stewed eggs, omelets, etc. The PS group was provided with ready-to-eat commercial options like egg white or chicken sausages. On average, WE participants received 800 to 850 kcal/day, 2100 to 2260 mg of dietary cholesterol, and 70 to 80 g of protein, while PS participants were given 810 to 850 kcal/day, 50 to 220 mg of dietary cholesterol, and 70 to 80 g of protein on school days. Control group participants received standard school lunches aligned with the Thai school lunch program. No group received additional meals or supplements on weekends. Throughout the study, participants underwent follow-up assessments at baseline, 14 weeks, and 35 weeks.

### 2.3. Primary Outcome

#### 2.3.1. Blood Test

A serum sample was obtained and analyzed for a complete blood count, transferrin, prealbumin, albumin, fasting blood sugar (FBS), lipid profiles, and IGF-1. These measurements were conducted at an accredited clinical laboratory (Siriraj Hospital, Bangkok, Thailand) using chemiluminescent immunoassays (CLIA) technique.

#### 2.3.2. Changes in IGF-1

Due to limited blood availability, in each group, 15% of all participants were randomly selected for serum IGF-1 measurements, based on the highest and lowest changes in height when compared between baseline and 35 weeks within each group (*n* = 48 for each group). These participants were evenly divided between females and males to homogenize the pooled samples. Subsequently, to assess the association between IGF-1 and SCFA, blood samples with IGF-1 results were matched with individuals who had SCFA reports.

#### 2.3.3. Gut Microbiota Analysis

Microbial DNA was isolated from 250 mg of feces using a QIAamp PowerFecal Pro DNA Kit (QIAGEN, Hilden, Germany). The samples were sent to the Centre d’expertise et de Services Génome Québec (Génome Québec, Montréal, QC, Canada) for 16S rRNA sequencing. The V4 region of the 16S rRNA gene was amplified using primer 515F–806R, reverse-barcoded: GTGCCAGCMGCCGCGGTAA/GGACTACHVGGGTWTCTAAT, according to the manufacturer’s protocols. AmpliconSeq sequencing was performed on the NovaSeq platform (Génome Québec, Montréal, QC, Canada) (detail in Supplement Method S1).

#### 2.3.4. Short-Chain Fatty Acid Measurement

Based on primary aims in the previous study which focused on children’s growth compared between baseline, 14 weeks, and 35 weeks of the intervention [3], the primary outcome of this study is focused on the comparison of short-chain fatty acid changes between baseline and 35 weeks (endpoint). Among all 635 participants, 197 were the control group, 200 were the PS group, and 238 were the WE group. For the in-depth analysis of microbial metabolites, feces samples were randomly collected from seventy-five participants (*n* = 25 per group). Participants were chosen based on two criteria: (1) the extent of height change between the baseline and 35 weeks of the intervention, and (2) the relative abundance change of *Bifidobacterium*. The focus was on both the lowest and highest changes in these two factors within each group. This stratified random selection process was guided by previously published data on the impact of egg supplementation on changes in *Bifidobacterium* [3]. The determination of participants per group was calculated considering a type I error rate (α) of 1% and a type II error rate (β) of 20%. An allowance for a possible dropout rate of 10% was taken in to enhance the statistical power.

Each stool sample, weighing one gram, was thoroughly mixed with 3 mL of internal standard solution (0.85 μM heptanoic acid) after thorough vortexing and subsequent centrifugation at 2000× *g* 4 °C for 10 min. Then, 10 µL of 1 M phosphoric acid was added to 300 µL of the resulting supernatant. The resulting supernatant was filtered using a syringe filter (0.45 µm) and transferred into a chilled GC vial. Finally, the vials were stored at −20 °C prior to the subsequent analysis.

The reaction mixture was analyzed using a GC- Flame Ionization Detector (GC-FID) on a model 7890A instrument (Agilent Technologies in Santa Clara, CA, USA) equipped with a capillary column (DB-FFAP, 30 m × 0.53 mm × 0.5 um). The initial oven temperature was set to 90 °C and held for 1 min, followed by an increase at a rate of 20 °C per minute until reaching 190 °C, where it was held for 25 min. The injector and detector temperatures were 210 °C, and the split vent purge was maintained at 20 mL per minute after 0.5 min. The gas flow and septum purge rates were 7.7 and 3.0 mL per minute, respectively. The H2 flow was 40 mL per minute, air flow was 400 mL per minute, and makeup flow was 45 mL per minute. The peak areas of the respective analyte production ions were measured against the peak areas of corresponding internal standard production ions for quantification. A standard SCFAs-phenol-p-cresol mixture containing acetic acid (acetate), propionic acid (propionate), butyric acid (butyrate), isobutyric acid (isobutyrate), valeric acid (valerate), isovaleric acid (isovalerate), caproic acid, hexanoic acids, phenol, and p-cresol (Sigma-Aldrich, Darmstadt, Germany) was utilized to determine the fatty acid concentration in µmol/mL by comparing their peak areas with the standard.

### 2.4. Dietary Assessment

Participants’ food consumption was tracked using a 3-day dietary record (one day for the weekend and two days for the week) [24] and a face-to-face food recall by specially trained nutritionists and dietitians. The energy and nutrient intakes recorded in each recall were added together to calculate the observed complementary feeding intakes. The Thai school lunch program (NECTEC) in Pathumthani, Thailand, managed the micronutrients and macronutrients to ensure consistency across schools. Lastly, the INMUCAL–Nutrient Software version 4.0 (INMU) from Nakhon Pathom, Thailand, was utilized to compute energy intake and nutrient values.

### 2.5. Statistical Analysis

In this prospective interventional study, a randomized controlled trial was undertaken with the primary aim of investigating the long-term effects of egg supplementation on growth and microbiota, as previously detailed [3]. We investigated the effect of egg supplementation on IGF-1 and gut microbiota-derived metabolites level as well as the association between gut microbiota, gut microbiota-derived metabolites, IGF-1, and dietary intake. Baseline and endpoint of IGF-1 levels were analyzed, alongside before-and-after measurements of all SCFAs including acetate, propionate, butyrate, isobutyrate, valerate, isovalerate, and total SCFA levels. For normalization purposes, the concentrations of each SCFA and the total SCFA were transformed. A Generalized Estimating Equation (GEE) was employed to estimate the mean changes of each SCFA from baseline to endpoint. Continuous variables were expressed as mean ± SEM. Demographic characteristics were evaluated using ANOVA and chi-squared tests. Prior to analysis, gut bacteria exhibiting high and low changes in relative abundance were ranked, and the top twenty displaying the lowest and highest relative abundance of microbiota in comparison to the baseline were selected for analysis. Pearson’s correlation was employed to examine the relationships between gut bacteria, gut microbiota-derived metabolites, growth hormone, and dietary variables. Statistical significance was defined as *p* < 0.05. All of the data analysis was performed using STATA version 17.0 (Stata Corporation, College Station, TX, USA), and graphical representations were created using GraphPad Prism (version 8.0.2).

## 3. Results

### 3.1. Baseline Characteristics

The participants’ characteristics are summarized in Table 1. The mean age of the participants was 9.7 ± 1.01 years, with an equal representation of males and females in a 1:1 ratio. The proportion of underweight, overweight, and obese participants was over 12%, 8%, and 13%, respectively. Furthermore, the proportion of stunted and tall-stature participants was 12% and 9%, respectively. The mean of the clinical data, including fasting blood sugar, transferrin, prealbumin, albumin, and lipid profile, are presented in Table 1. No significant differences were observed between the groups. Nutritional intake was assessed during the first 4 weeks of the intervention. Dietary information was not collected before the intervention started due to a school break. Notably, significant differences in cholesterol intake were noted among the WE group (302.6 ± 115.90 mg/day), the PS group (209.8 ± 116.69 mg/day), and the control group (182.3 ± 90.72 mg/day). However, no significant differences in total energy intake, protein, carbohydrate, fat, saturated fatty acid, or fiber were observed among the three groups.

### 3.2. Change in IGF-1 Level

**Figure 1:**
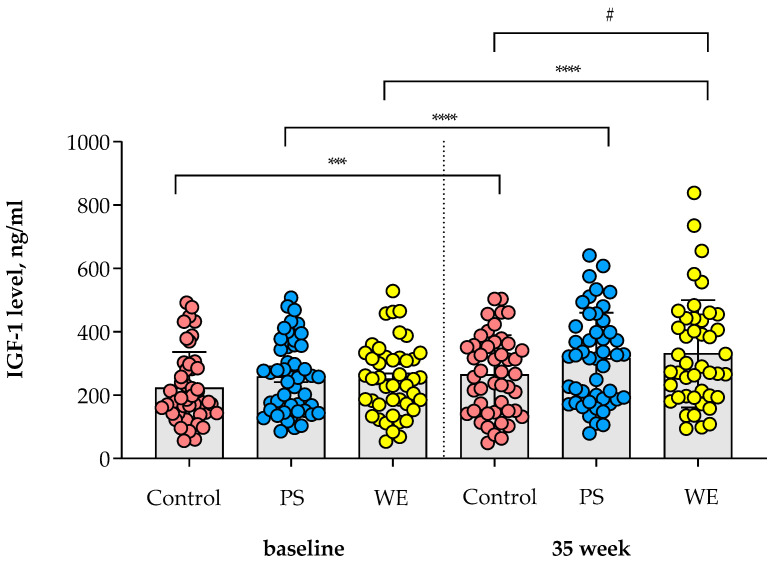
Changes in IGF-1 levels within the study groups. The IGF-1 levels of the child subjects were compared both between groups and from baseline to the 35th week of the follow-up period. The bar graph represents the means, while the error bars indicate standard error of the mean (SEM). *** indicates statistical significance within group at *p* < 0.001. **** indicates statistical significance within group at *p* < 0.0001. ^#^ indicates statistical significance between groups at *p* < 0.05. Abbreviations; PS = protein substitute group; WE = whole egg group; IGF-1 = insulin-like growth factor 1.

The results indicated a significant increase (*p* < 0.05) in IGF-1 levels at 35 weeks in the WE group (330.9 ± 25.5 ng/mL) compared to the Control group (264.3 ± 18.2 ng/mL), while no significant difference was observed in the PS group (315.3 ± 21.2 ng/mL) (ns). Upon furthermore, in comparison to the baseline, the IGF-1 levels in the WE group were elevated by 79.4 ± 13.4 ng/mL (*p* < 0.0001), whereas the PS group and Control group showed increases of 57.4 ± 12.1 ng/mL (*p* < 0.0001) and 41.7 ± 9.8 ng/mL (*p* = 0.0001), respectively (see Figure 1). Consequently, the supplementation of whole eggs resulted in a significant improvement in growth hormone levels compared to the control.

### 3.3. Change in SCFAs from Baseline to Endpoint

**Figure 2:**
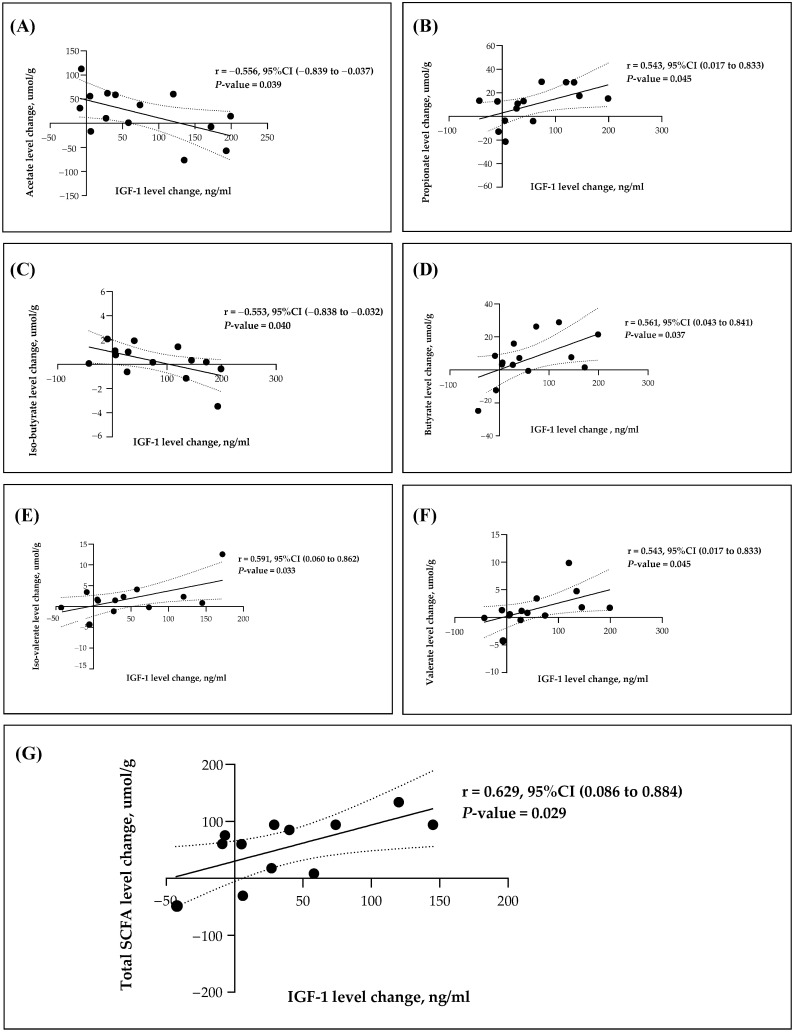
Pearson’s coefficient between changes in each SCFA and IGF-1 levels. This figure illustrates the correlation between changes in SCFAs and IGF-1 levels. The comparison involves the alterations in SCFA and IGF-1 values between the baseline and week 35. (**A**) Correlation between changes in acetate and IGF-1; (**B**) Correlation between changes in propionate and IGF-1; (**C**) Correlation between changes in isobutyrate and IGF-1; (**D**) Correlation between changes in butyrate and IGF-1; (**E**) Correlation between changes in isovalerate and IGF-1; (**F**) Correlation between changes in valerate and IGF-1; (**G**) Correlation between changes in total SCFA and IGF-1. The black points indicate observations. The solid line represents predictions, while the dashed lines indicate the 95% CI. Abbreviation: SCFA, short-chain fatty acids; IGF-1, insulin-like growth factor 1.

SCFAs are a group of fatty acids that have a relatively small number of carbon atoms in their molecular structure. They are produced by the fermentation of dietary fibers by gut bacteria in the colon. SCFAs play important roles in gut health and overall metabolism, as they can provide energy to colon cells, influence immune function, and affect various physiological processes. Three SCFAs typically produced in larger quantities by gut microbiome are acetate, propionate, and butyrate and in smaller quantities are isobutyrate, isovalerate, valerate, and hexanoic acid [18]. The relative abundance of these SCFAs can vary based on factors such as diet, gut microbiota composition, and overall gut health [25]. To compare the relationship between SFCAs and IGF-1, we, therefore, compared the changing values of SCFAs between baseline and week 35 in the sample participants of all groups and compared them with the changing values of IGF-1 between baseline and week 35 (Figure 2). An association was found between IGF-1 and gut microbiome metabolites. Notably, IGF-1 levels exhibited a significantly positive correlation with propionate, butyrate, isovalerate, valerate, and total SCFA. Conversely, these levels demonstrated a negative correlation with acetate and isobutyrate, as shown in Figure 2.

The concentration of SCFAs is graphically presented in Supplement Figure S1, and a detailed tabulation of these concentrations is presented in Table 2. At 35 weeks of study, a statistically significant upsurge in propionate levels was observed in the PS group (8.10 ± 3.83 umol/g). In contrast, a reduction in propionate concentrations was noted in the WE group (−3.62 ± 3.75 umol/g) compared to the Control group (1.90 ± 3.75 umol/g) (*p* < 0.033). However, there were no significant disparities in acetate, butyrate, and three other common metabolites, including isobutyrate, valerate, isovalerate, among the three groups. Notably, acetate levels exhibited elevations within both the PS and Control groups, approximating values of 13.64 ± 9.35 and 13.05 ± 9.35 umol/g, respectively. Conversely, the WE group displayed a more modest rise, reaching 2.43 ± 9.35 umol/g in comparison to the baseline values. The observed fluctuation in acetate concentration was less pronounced in the WE group relative to the PS and Control groups. The concentration of butyrate demonstrated an upward inclination within the PS group (0.08 ± 2.23 umol/g) while recording decreases in both the WE and Control groups (−1.68 ± 2.23 umol/g and −0.73 ± 2.23 umol/g, respectively), when compared with the baseline measurements.

Meanwhile, isobutyrate decreased in all three groups, whereas isovalerate decreased in the PS and the Control groups (−0.86 ± 0.63 and −0.80 ± 0.61 μmol/g, respectively) but increased in the WE group (0.79 ± 0.63 μmol/g), compared to baseline. Additionally, valerate increased in the WE group (0.19 ± 0.45 μmol/g) and the PS group (0.35 ± 0.45 μmol/g), but not the Control group, as it was decreased. Finally, total SCFA concentrations remained stable in the WE group, while they increased in the PS and Control groups. The PS group showed better attainment of a higher total SCFAs concentration compared to the WE and Control groups. However, we observed that concentrations of hexanoic acid, phenol, and p-cresol increased in the WE group but decreased in the PS group, compared to baseline. These specific phenol and p-cresol compounds derive from the metabolism of aromatic amino acids, such as tyrosine, phenylalanine, and tryptophan, originating from dietary proteins. In summary, the supplementation of whole eggs demonstrated the potential to preserve the constancy of gut microbiota metabolite levels yet concurrently appeared to heighten the precursors of potentially adverse metabolites, including phenol and p-cresol, also recognized as uremic toxins.

### 3.4. Association between SCFAs and Gut Microbiota

The relative abundance of the 42 genera of gut microbiota in each group was assessed, with the top 20 genera having the highest and lowest percentage changes, along with common bacteria identified in our previous report [3]. These genera were classified into the phyla Actinobacteria, Firmicutes, Proteobacteria, Euryarcheotic, and Bacteroidetes. The Control group comprised fifteen bacteria, whereas the PS and WE groups had thirteen bacteria each, which exhibited a significant correlation with SCFAs (Table 3 and Supplement Figure S2).

**Figure 3:**
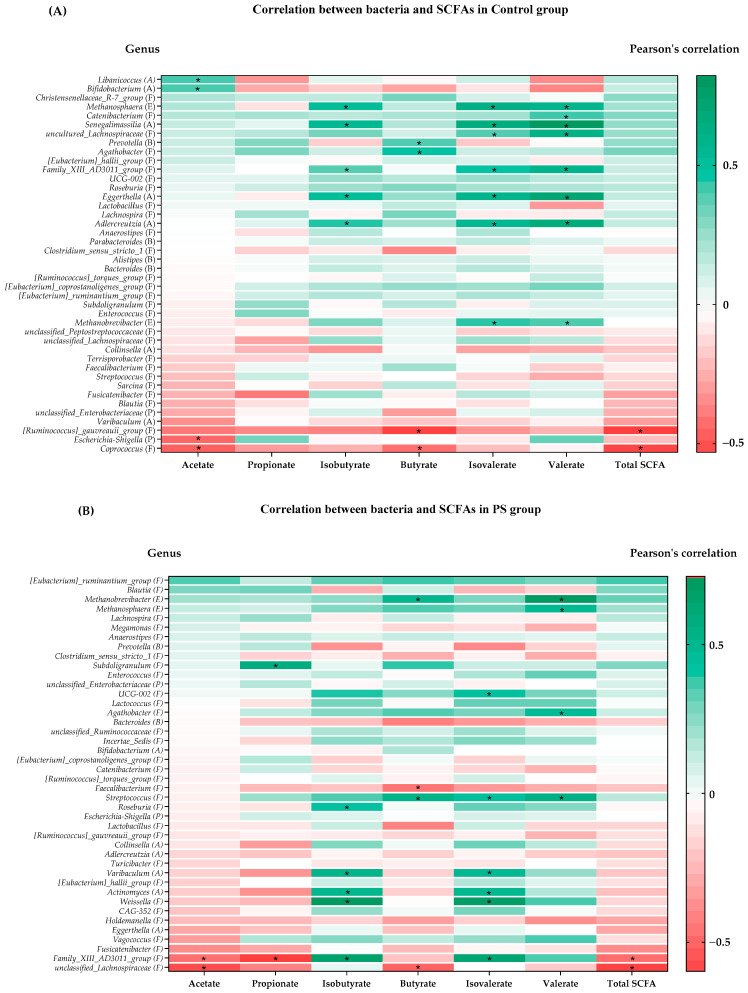

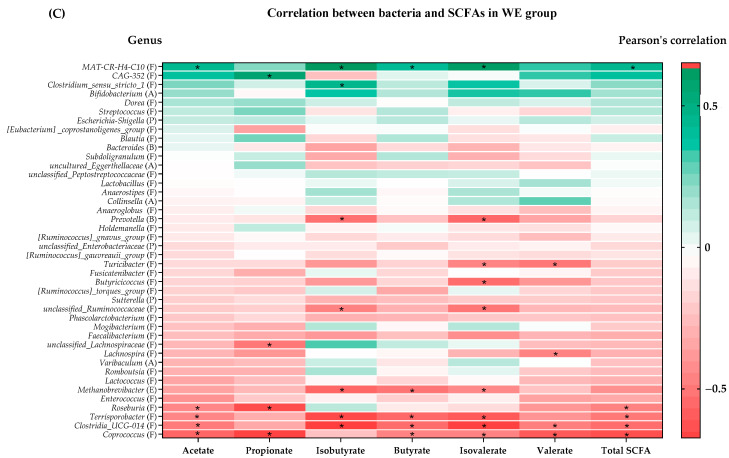
Heatmap of Pearson’s coefficient between top 42 differential abundance of gut microbiota and concentrations of SCFAs. (**A**) Control group (**B**) PS group (**C**) WE group. The bacterial genus is sorted from negative (brick red squares) to insignificant (white squares) to positive (brick green squares) correlations. Significant correlations are marked by * (correlation coefficient ≥ ±0.3). Abbreviations: (A) = Actinobacteriota; (F) = Firmicutes; (C) = Cyanobacteria; (P) = Proteobacteria; (E) = Euryarchaeota; (B) = Bacteroidota.

As depicted in Figure 3A, in the Control group, a substantial proportion of the bacteria correlating with isobutyrate, isovalerate, and valerate belonged to the phyla Firmicutes and Actinobacteria. Genera such as *Senegalimassilia, Methanosphaera, Eggerthella, Adlercreutzia,* and *Fammily_XIII_AD3011_group* exhibited a significantly positive correlation with isobutyrate, isovalerate, and valerate. Moreover, *uncultured Lachnospiraceae* and *Methanbrevibacter* genera showed a significant positive correlation with isovalerate and valerate. The most robust positive correlations were observed with *Senegalimassilia* genera for isobutyrate [r = 0.566 95%CI (0.221 to 0.786), *p* = 0.003], isovelerate [r = 0.674 95%CI (0.379 to 0.844), *p* = 0.0002] and valerate [r = 0.822 95%CI (0.633 to 0.919), *p* < 0.0001, respectively]. Moreover, *Libanicoccus* and *Bifidobacterium* genera demonstrated a significant positive correlation with acetate [r = 0.430 95%CI (0.042 to 0.705), *p* = 0.032 and r = 0.428 95%CI (0.039 to 0.704), *p* = 0.033, respectively], while *Prevotella* and *Agathobacter* genera showed a significantly positive correlation with butyrate [r = 0.412 95%CI (0.020 to 0.694), *p* = 0.041 and r = 0.494 95%CI (0.123 to 0.744), *p* = 0.012, respectively]. Conversely, *Coprococcus* genera exhibited a significant negative correlation with acetate (*p* = 0.028), butyrate (*p* = 0.046), and total SCFA (*p* = 0.009). In addition, *Escherichia-Shigella* genera had a significant negative correlation with acetate, and *[Runminococus]_gauvreauii_group* genera displayed a significant negative correlation with isobutyrate and total SCFA.

As shown in Figure 3B, among the PS group, genera such as *Varibaculum*, *Actinomyces*, *Weissella*, and *Family_XIII_AD3011_group* genera exhibited a significant positive correlation with isobutyrate and isovalerate. Furthermore, the *Roseburia* genera demonstrated a significant positive correlation with isobutyrate, while *Streptococcus* and *UCG-002* genera displayed a significant positive correlation with isovalerate. Notably, *Weissella* genera displayed the most robust positive correlation with isobutyrate [r = 0.729 95%CI (0.443 to 0.880), *p* = 0.001] and isovalerate [r = 0.694 95%CI (0.385 to 0.863) *p* = 0.0003]. A significant proportion of bacteria correlating with valerate were from the phyla Euryarchaeota (*Methanobrevibacter* and *Methanosphaera*) and Firmicutes (*Agathobacter* and *Streptococcus*). The strongest positive correlation with valerate was found to be *Methanobrevibacter* [r = 0.717 95%CI (0.424 to 0.874), *p* = 0.0002]. Additionally, *Methanobrevibacter* and *Streptococcus* genera exhibited a significant positive correlation with butyrate [r = 0.521 95%CI (0.127 to 0.773), *p* = 0.013 and r = 0.549 95%CI (0.166 to 0.788) *p* = 0.008, respectively]. Notably, only one *Subdoligranulum* genera exhibited a significant positive correlation with propionate [r = 0.549 95%CI (0.230 to 0.812), *p* = 0.004]. Moreover, bacteria exhibited a negative correlation with SCFA including *Faecalibacterium* and *unclassified_Lachnospira* genera, which exhibited a significant negative correlation with butyrate [r = −0.435 95%CI (−0.724 to −0.017), *p* = 0.043 and r = −0.489 95%CI (−0.755 to −0.085), *p* = 0.021, respectively]. Interestingly, *Family_XIII_AD3011_group* and *unclassified_Lachnospira* genera belonging to the phyla Firmicutes displayed a significant negative correlation with major metabolites (acetate, propionate, butyrate, and total SCFA), while exhibiting a positive correlation with two other common metabolites, including isobutyrate and isovalerate.

In Figure 3C, within the WE group, it was observed that the *MAT-CR-H4-C10* genera within the phyla Firmicutes displayed the strongest positive correlation with acetate [r = 0.452 95%CI (0.038 to 0.734), *p* = 0.035], isobutyrate [r = 0.652 95%CI (0.318 to 0.842), *p* = 0.001], butyrate [r = 0.440 95%CI (0.022 to 0.727), *p* = 0.041], isovalerate [r = 0.622 95%CI (0.271 to 0.827), *p* = 0.002], and total SCFA [r = 0.463 95%CI (0.0516 to 0.740), *p*= 0.030]. Additionally, the *Clostridium_sensu_stricto_1* genus displayed a significant positive correlation with isobutyrate, and the *CAG-352* genera exhibited a positive correlation with propionate [r = 0.592 95%CI (0.227 to 0.811), *p* = 0.004]. Bacteria from phyla Euryarchaeota (*Methanobrevibacter*) exhibited a significant negative correlation with isobutyrate, butyrate, and isovalerate. Moreover, the *Prevotella* and *unclassified_ Ruminococcus* genera demonstrated a significant negative correlation with isobutyrate. Interestingly, nearly all the bacteria within the phyla Firmicutes, including *Roseburia*, *Terrisporobacter*, *Clostridia_UCG-014*, and *Coprococcus* genera, exhibited a significantly negative correlation with SCFA metabolites, especially acetate and total SCFA. In summary, both the Firmicutes and Actinobacteriota displayed a strong association with gut microbiota-derived metabolites.

According to the changes in SCFAs concentrations presented in Table 2 and Figure 3, we identified that the PS group exhibited the most significant increase in acetate from baseline among the three groups. Table 3 also summarizes the relationship between SCFA-producing bacteria and SCFAs levels in each group. The findings indicate that the *MAT-CR-H4-C10*, *Libanicoccus* [26], and *Bifidobacterium* genera displayed a significant positive correlation with acetate production, a documented healthy gut homeostasis pathway during carbohydrate fermentation in the colon [27]. Notably, a prior study indicated a 1.2-fold increase in the relative abundance of *Bifidobacterium* within the WE group compared to baseline, whereas the *MAT-CR-H4-C10* and *Libanicoccus* genera showed a decreased relative abundance. This suggests that the impact of *Bifidobacterium* genera on acetate production might be relatively lower than that of the *MAT-CR-H4-C10* genera, which demonstrated the most robust positive correlation with acetate. Furthermore, within the WE group, the genera *Roseburia*, *Terrisporobacter*, *Clostridia_UCG-014*, and *Coprococcus,* all belonging to the Firmicutes phylum, exhibited an increase in relative abundance from baseline and were negatively correlated with acetate. Notably, *Coprococcus* genera displayed the most pronounced negative correlation [r = −0.544 95%CI (−0.785 to −0.158), *p* = 0.009]. Hence, these bacteria might have an influence on acetate concentration, particularly in the WE and the Control groups when compared to the PS group.

Interestingly, at the 35-week follow up, propionate concentration increased in the PS and Control groups but decreased in the WE group. The results show that propionate-producing bacteria, including two genera, *CAG-352* and *Subdoligranulum*, exhibited significant positive correlations with propionate in the WE and PS groups, respectively. Furthermore, changes in the relative abundance of bacteria indicated an increase from baseline for *CAG-352* and *Subdoligranulum* in the PS group, whereas a decrease was observed in the WE group. This suggests that these bacteria could potentially impact propionate levels. Conversely, the butyrate content among the three groups remained relatively stable, consistent with the correlation data. Butyrate-producing bacteria displayed minor changes in the relative abundance within the gut microbiota, resulting in the absence of a noticeable trend in butyrate level changes.

Finally, total SCFA content, encompassing acetate, propionate, and butyrate, demonstrated relative stability within the WE group when compared to the PS and Control groups. Although there was an increasing trend from baseline, it did not achieve statistical significance. In summary, the Firmicutes phylum (*MAT-CR-H4-C10, CAG-352*, and *Subdoligranulum*) and the Actinobacteriota phylum (*Libanicoccus* and *Bifidobacterium*) exhibited a significant positive correlation with total SCFAs. Conversely, the major bacterial genera negatively correlated with total SCFAs mostly belonged to the Firmicutes phylum (*Roseburia*, *Terrisporobacter*, *Clostridia_UCG-014*, and *Coprococcus*), with *Coprococcus* demonstrating the most potent negative correlation.

### 3.5. Association between SCFAs and Nutrition Composition

**Figure 4:**
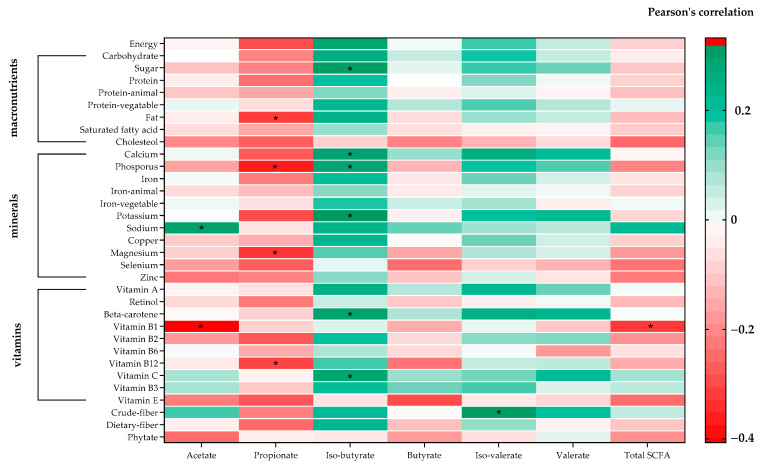
Heat map of Pearson’s coefficient between SCFA and nutrients. The nutrient composition is sorted from negative (red) to positive (green) correlations, assessed by Pearson’s correlation. Significant correlations are marked by * (correlation coefficient ≥ ±0.3).

The correlation between SCFAs and nutritional composition is depicted in Figure 4. Among macronutrients, acetate and propionate metabolites exhibited no significant correlations, except for isobutyrate, butyrate, isovalerate, and valerate, which displayed a trend toward association. Sugar consumption displayed a positive association with isobutyrate (r = 0.318, 95%CI 0.060 to 0.536, *p* = 0.017), while fat intake demonstrated a negative association with propionate (r = −0.307, 95%CI −0.528 to −0.048, *p* = 0.021). Interestingly, we observed negative associations between cholesterol and vitamin E intake with all SCFAs (non-significant). Furthermore, nearly all micronutrients (including minerals and vitamins) exhibited negative correlations with acetate, propionate, and total SCFA. Specifically, phosphorus, magnesium, and vitamin B12 intake displayed a statistically significant negative correlation with propionate, whereas a positive correlation was observed with isobutyrate, isovalerate, and valerate. Notably, calcium, phosphorus, potassium, beta-carotene, and vitamin C exhibited a statistically significant positive correlation with isobutyrate. On the other hand, sodium consumption exhibited a significant correlation with acetate (r = 0.307, 95%CI 0.048 to 0.527, *p* = 0.021), with vitamin C, vitamin B3, and crude fiber showing a trend toward association. In summary, nearly all of the nutritional intake, encompassing both macronutrients and micronutrients, displayed associations with gut microbiome metabolites, particularly isobutyrate and isovalerate, which are branched-chain fatty acids. The observed negative or positive correlations of macronutrients and micronutrients suggest that alterations in metabolites important to the SCFA fermentation process may impact the pattern of intestinal fermentation. This resulted in different SCFA concentrations in each group.

## 4. Discussion

The intricate influence of gut microbiota on host health and physiological processes has garnered increasing attention, particularly regarding the impact of microbial metabolites. In this study, we investigated the relationship between SCFAs derived from gut microbiota and growth hormone levels in relation to long-term egg supplementation in primary school students. Our aim is to display the potential interplay between dietary egg interventions, gut microbiota, and host physiology. Our findings suggest that long-term supplementation with whole eggs leads to an increase in circulating IGF-1 levels, which could potentially contribute to both bone formation and skeletal growth. This discovery aligns with previous studies [3], where the inclusion of whole eggs resulted in improved growth, particularly in terms of height. However, our results showed that IGF-1 level was significantly elevated in the control group after 35 weeks. The investigation focused on children facing the risk of malnutrition in rural settings, revealing noteworthy considerations regarding family income and consistent access to nourishing meals, both pivotal factors influencing growth. Typically, primary school children benefit from nutritionally comprehensive school lunches as part of the Thai school lunch program, coupled with milk provision throughout the school term. Consequently, growth hormone levels experienced an upswing across all groups, including the control group. Notably, the group subjected to whole egg supplementation exhibited higher growth hormone levels in comparison to their counterparts in the other groups. Several studies have suggested that the production of IGF-1 can be stimulated by nutritional factors, particularly optimal protein, and caloric intake, which can significantly influence IGF-1 levels. Clinical evidence demonstrates that higher energy, protein, and milk intakes are associated with elevated levels of IGF-1 [28,29]. Similarly, Switkowski et al. reported that protein intake during early childhood may play a role in activating IGF-1 [30]. Thus, these data confirm that a high-quality protein intake and adequate energy intake are positively correlated with the linear growth of children.

We identified a positive correlation between circulating IGF-1 and all SCFAs, except for acetate. However, it remains uncertain whether these correlations extend to microbiota metabolites, and the underlying mechanism of this association remains unexplored. Recent data have shown that microbiota-metabolites, especially SCFAs, can induce IGF-1, indicating a potential mechanism through which the microbiota may impact bone health [31]. Likewise, Li et al. reported a positive correlation between SCFAs, including butyrate, and serum IGF-1 levels in the feces of children [32].

In addition, our findings showed that the overall content of SCFA, including acetate, propionate, and butyrate, remained relatively consistent with whole egg supplementation. Concerning the results regarding the relationship between gut microbiota and SCFAs production following 35 weeks of whole egg supplementation, among the 42 gut bacteria that displayed changes in relative abundance from the lowest and highest analyses in this study, *MAT-CR-H4-C10*, *CAG-352*, and *Clostridium_seu_stricto_1* exhibited the strongest positive correlations with all SCFAs. In contrast, *Roseburia*, *Terrisporobacter*, *Clostridia_UCG-014*, and *Coprococcus* showed the most pronounced negative correlations, although we observed an increase in the abundance of *Bifidobacterium* and *Prevotella*. Interestingly, the increase in *Bifidobacterium* following 35 weeks of whole egg supplementation in undernourished children effectively boosted the quantity of beneficial bacteria without affecting SCFA levels. This highlights the multifaceted nature of microbial metabolite production in the gut, which may be influenced by various factors. Similarly, a review study by Wong et al. suggests that the rate and amount of SCFA production depend on the numbers and types of microflora present in the colon, substrate source, and gut transit time [33].

Nevertheless, the emerging scientific literature has established a connection between reduced *Bifidobacterium* counts and various disorders. Diminished relative abundances of *Bifidobacterium* species have been associated with conditions such as antibiotic-associated diarrhea, irritable bowel syndrome (IBS), inflammatory bowel disease (IBD), obesity, allergies, and regressive autism [34,35]. *Bifidobacterium* species perform a range of diverse functions, including the synthesis of B vitamins, antioxidants, polyphenols, and conjugated linoleic acids. They also play critical roles in early-life immune system development, maintenance of immune homeostasis, the preservation of gut barrier functions, protection against pathogens through acid production and bacteriocin release, and the inhibition of pathogen adhesion [36,37]. However, these functions are strain-specific rather than universal across the *Bifidobacterium* genus or specific species. Another pivotal role involves the production of acetate and lactate during carbohydrate fermentation, which contributes significantly to gut homeostasis and overall host health.

The microbiome holds the potential to significantly influence the host’s absorption of various dietary nutrients, thereby indirectly impacting the physiology of micronutrients [38]. Certain strains of microbes are known to synthesize vitamins and cofactors, and emerging evidence suggests that microbial metabolites can impact the metabolic and physiological pathways of micronutrients within the human body [39]. Moreover, the production of vitamins and cofactors by microbes can serve as vital nutrients for colonocytes, fostering competition with harmful microorganisms, and modulate immune responses [40]. Additionally, because bacteria can alter the efficiency of bile acids in emulsifying dietary lipids and forming micelles, the microbiome might also play a role in the absorption of lipid-soluble vitamins [41].

Several factors, including the physicochemical properties of foods, nutrient availability, colonic transit time, and the host’s age, can potentially influence how diet affects the colonic microbiota [42]. Interestingly, even substances not typically classified as “prebiotics”, such as phytochemicals, have the potential to modify microbial composition and function. Vitamins and minerals also fall into this class of substances, but their impact on the gut microbiome is not yet well-established or fully understood [43]. This may be partly due to the fact that a significant portion of vitamins are absorbed in the upper small intestine, resulting in relatively low concentrations of vitamins or minerals reaching the distal segments of the gastrointestinal tract [43].

This research boasts several strengths. To the best of our knowledge, it represents the first long-term study of continuous egg supplementation and its effects on gut metabolites such as SCFAs in malnourished children. This suggests that eggs, in addition to conventional protein sources like milk, can serve as a valuable option for supplementation to effectively stimulate the production of gut bacteria metabolites. Another notable strength lies in our identification of a connection between growth factors and the end products of the gut microbiota, potentially shedding light on an indirect link to improved growth.

Nevertheless, this study does have certain limitations. Firstly, whole egg supplementation was provided during school breaks, making it difficult to control additional egg consumption by participants in both the protein substitute group and the control group during weekends and school breaks. Secondly, the time during transportation and sample extraction can impact the concentration of gut metabolite production. Ideally, the optimal timeframe for extraction should be within 1 h or a maximum of 24 h [44]. However, in this study, we collected samples from participants in rural primary schools, where the transportation time to the laboratory center exceeded 3 h. This extended travel time may have contributed to changes in gut metabolite concentrations. Third, the study employed a cross-sectional design, with health outcomes tracked for one year following continuous egg supplementation. Notably, no data related to infections or illnesses were collected. It is imperative to consider illness data, as it directly influences the type, prevalence, and quantity of bacteria capable of producing metabolites during that time frame.

## 5. Conclusions

In conclusion, this study contributes to our understanding of the interplay between dietary interventions, gut microbiota, SCFAs, and growth factors. The positive impact of whole egg supplementation on IGF-1 levels suggests a potential avenue for promoting bone health and growth in children. The associations between SCFAs and specific gut microbiota genera highlight the intricate connections between diet, microbiota, and host health. As research in this field advances, it promises to provide valuable insights into optimizing health through dietary interventions that harness the power of the gut microbiota and its metabolites.

## Figures and Tables

**Table 1:** Baseline characteristics of participants.

Variables		Control [*n* = 26]	PS [*n* = 25]	WE [*n* = 24]	*p*-Value
		*n* (%)	*n* (%)	*n* (%)	
Age, mean (SD), year		9.7 (1.15)	9.8 (0.93)	9.7 (0.96)	0.948
Sex					0.841
	Male	12 (46.15)	13 (52.00)	13 (54.17)	
	Female	14 (53.85)	12 (48.00)	11 (45.83)	
Weight, mean (SD), kg		37.1 (16.23)	32.3 (10.56)	32.5 (8.03)	0.298
Height, mean (SD), cm		139.3 (9.21)	138.5 (11.20)	138.7 (7.92)	0.958
Obesity status					0.829
	Underweight	3 (11.5)	3 (12.0)	3 (12.5)	
	Normal	15 (57.6)	18 (72.0)	17 (70.8)	
	Overweight	3 (11.5)	2 (8.0)	1 (4.2)	
	Obese	5 (19.2)	2 (8.0)	3 (12.5)	
Height status					0.386
	Stunted	3 (11.5)	4 (16.0)	2 (8.3)	
	Normal	20 (76.9)	18 (72.0)	21 (87.5)	
	Tall stature	3 (11.5)	3 (12.0)	1 (4.2)	
Blood pressure, mean (SD), mm Hg					
	Systolic	102.3 (10.93)	104.0 (11.42)	98.7 (7.35)	0.178
	Diastolic	69.1 (6.14)	71.2 (5.80)	69.7 (6.16)	0.447
Fasting blood sugar, mean (SD), mmol/L		88.6 (10.34)	86.4 (7.58)	87.9 (8.95)	0.674
Transferrin, mean (SD), g/L		256.6 (20.50)	264.1 (29.98)	262.8 (33.29)	0.601
Prealbumin, mean (SD), μmol/L		0.2 (0.03)	0.2 (0.04)	0.2 (0.04)	0.314
Albumin, mean (SD), g/L		4.4 (0.24)	4.4 (0.23)	4.4 (0.25)	0.579
Blood lipid level, mean (SD), mmol/L					
	TC	176.2 (30.24)	176.2 (31.97)	185 (31.00)	0.521
	TG	84.9 (23.24)	80.2 (35.86)	82.9 (30.10)	0.855
	HDL-C	55.5 (11.61)	57.8 (9.84)	57.5 (10.05)	0.710
	LDL-C	103.6 (25.60)	102.3 (27.53)	110.8 (25.61)	0.477
Nutrition intake, mean (SD)					
	Total energy intake, kcal/day	951.1 (233.95)	863.9 (263.33)	749.7 (268.36)	0.110
	Protein intake, g/day	37.8 (9.08)	39.9 (12.98)	35.0 (9.50)	0.455
	Carbohydrate, g/day	99.2 (22.58)	92.9 (41.17)	86.1 (42.34)	0.287
	Fat, g/day	34.6 (11.34)	36.9 (15.76)	29.5 (9.75)	0.262
	Saturated fatty acid, g/day	7.7 (3.45)	8.3 (2.69)	7.1 (3.44)	0.127
	Cholesterol, mg/day	182.3 (90.72) ^b^	209.8 (116.96) ^b^	302.6 (115.90) ^a^	0.011
	Dietary fiber, g/day	3.6 (1.87)	3.3 (1.97)	2.8 (1.43)	0.454

Abbreviations: PS, protein substitute group; WE, whole egg group; TC, total cholesterol; TG, triglyceride; HDL-C, high-density lipoprotein cholesterol; LDL-C, low-density lipoprotein cholesterol. The nutrition intake data represent the mean difference between the intervention diets over the entire period of the intervention. ^a,b^ Values in the same row with the same superscript letters are significantly different among the groups at *p* < 0.05.

**Table 2:** SCFAs change in study group.

Parameters		Control [*n* = 26]	PS [*n* = 25]	WE [*n* = 24]	*p*-Value
		Mean ± SEM	Mean ± SEM	Mean ± SEM	
Acetate, μmol/g					
	baseline	75.16 ± 8.63	84.03 ± 8.63	72.98 ± 8.63	0.633
	week 35	88.21 ± 10.08	97.67 ± 10.08	75.41 ± 10.08	0.299
*p*-value		0.135	0.266	0.771	
Propionate, μmol/g					
	baseline	27.83 ± 3.57	31.11 ± 3.64	28.01 ± 3.57	0.732
	week 35	29.73 ± 3.93 ^ab^	39.21 ± 4.01 ^a^	24.39 ± 3.93 ^b^	0.033
*p*-value		0.644	0.102	0.385	
Isobutyrate, μmol/g					
	baseline	2.58 ± 0.46	2.62 ± 0.44	2.74 ± 0.46	0.931
	week 35	2.17 ± 0.35	2.54 ± 0.33	2.68 ± 0.35	0.482
*p*-value		0.297	0.845	0.891	
Butyrate, μmol/g					
	baseline	21.86 ± 2.17	21.96 ± 2.17	20.30 ± 2.17	0.832
	week 35	21.13 ± 2.29	22.04 ± 2.29	18.62 ± 2.29	0.551
*p*-value		0.752	0.976	0.602	
Isovalerate, μmol/g					
	baseline	3.92 ± 0.68	4.36 ± 0.70	4.01 ± 0.70	0.894
	week 35	3.12 ± 0.55	3.50 ± 0.56	4.80 ± 0.56	0.082
*p*-value		0.198	0.250	0.388	
Valerate, μmol/g					
	baseline	3.09 ± 0.50	2.62 ± 0.52	2.54 ± 0.51	0.704
	week 35	2.49 ± 0.38	2.97 ± 0.39	2.73 ± 0.39	0.674
*p*-value		0.384	0.556	0.525	
Hexanoic acid, μmol/g					
	baseline	0.46 ± 0.17	0.28 ± 0.18	0.65 ± 0.15	0.948
	week 35	0.36 ± 1.11	0.30 ± 1.20	2.15 ± 0.98	0.426
*p*-value		0.449	0.900	0.375	
Phenol, μmol/g					
	baseline	0.22 ± 0.36	0.82 ± 0.36	0.19 ± 0.36	0.757
	week 35	0.19 ± 2.20	0.57 ± 2.20	3.92 ± 2.20	0.681
*p*-value		0.192	0.649	0.499	
p-cresol, μmol/g					
	baseline	0.66 ± 0.15 ^ab^	0.97 ± 0.14 ^a^	0.44 ± 0.15 ^b^	0.022
	week 35	0.86 ± 0.16	0.91 ± 0.16	0.73 ± 0.17	0.752
*p*-value		0.108	0.564	0.017	
Total SCFA, μmol/g					
	baseline	135.11 ± 13.18	148.03 ± 13.18	130.67 ± 13.18	0.628
	week 35	147.79 ± 14.88	167.37 ± 14.88	129.88 ± 14.88	0.212
*p*-value		0.289	0.275	0.958	

Abbreviation: PS, protein substitute group; WE, whole egg group. ^a,b^ Values in the same row with the same superscript letters are significantly different among the groups at *p* < 0.05.

**Table 3:** Summary of SCFA-producing bacteria and SCFA changes in each group after 35-week intervention.

Groups	SCFA Levels Change	Bacterial Genera (Phylum)	Bacterial Genera Producing SCFAs [% Change in Relative Abundance from Baseline]	Significant Correlation of SCFAs (*p* < 0.05)
WE	↑ acetate ↓ propionate	Firmicutes	↓ *MAT-CR-H4-C10*	(+) acetate, isobutyrate, butyrate, iso-valerate, total SCFAs
	↓ butyrate		↓ *CAG-352*	(+) propionate
	Stability of total SCFAs		↓ *Clostridium_sensu_stricto_1*	(+) isobutyrate
			↓ *Butyricicoccus*	(−) isovalerate
			↓ *unclassified_Ruminococcaceae*	(−) isobutyrate, isovalerate
			↓ *unclassified_Lachnospiraceae*	(−) propionate
			↓ *Lachnospira*	(−) valerate
			↑ *Turicibacter*	(−) isovalerate, valerate
			↑ *Roseburia*	(−) acetate, propionate, total SCFAs
			↑ *Terrisporobacter*	(−) acetate, isobutyrate, butyrate, isovalerate, total SCFAs
			↑ *Clostridia_UCG_014*	(−) acetate, isobutyrate, butyrate, isovalerate, valerate, total SCFAs
			↑ *Coprococcus*	(−) acetate, propionate, butyrate, isovalerate, valerate, total SCFAs
		Bacteroidota	↓ *Prevotella*	(−) isobutyrate, isovalerate
		Euryarchaeota	↑ *Methanobrevibacter*	(−) isobutyrate, butyrate, isovalerate
PS	↑ acetate	Firmicutes	↓ *Streptococcus*	(+) butyrate, isovalerate, valerate
	↑ propionate		↓ *Weissella*	(+) isobutyrate, isovalerate
	↑ butyrate ↑ total SCFA		↓ *Family_XIII_AD3011_group*	(+) isobutyrate, isovalerate (−) acetate, propionate, total SCFAs
			↑ *Subdoligranulum*	(+) propionate
			↑ *UCG-002*	(+) isovalerate
			↑ *Agathobacter*	(+) valerate
			↑ *Faecalibacterium*	(−) butyrate
			↑ *Roseburia*	(+) isobutyrate
			↑ *unclassified_Lachnospiraceae*	(−) acetate, butyrate, total SCFAs
		Actinobacteriota	↓ *Varibaculum*	(+) isobutyrate, isovalerate
			↓ *Actinomyces*	(+) isobutyrate, isovalerate
		Euryarchaeota	↑ *Methanobrevibacter*	(+) butyrate, valerate
			↑ *Methanosphaera*	(+) valerate
Control	↑ acetate	Firmicutes	↓ *Catenibacterium*	(+) valerate
	↑propionate		↓ *uncultured_Lachnospiraceae*	(+) isovalerate, valerate
	Stability of butyrate		↓ *Family_XIII_AD3011_group*	(+) isobutyrate, isovalerate, valerate
	↑ total SCFA		↓ *[Ruminococcus]_gauvreauii_group*	(−) butyrate, total SCFAs
			↑ *Agathobacter*	(+) butyrate
			↑ *Coprococcus*	(+) acetate, butyrate, total SCFAs
Control		Actinobacteriota	↓ *Libanicoccus*	(+) acetate
			↓ *Bifidobacterium*	(+) acetate
			↓ *Senegalimassilia*	(+) isobutyrate, isovalerate, valerate
			↓ *Eggerthella*	(+) isobutyrate, isovalerate, valerate
			↓ *Adlercreutzia*	(+) isobutyrate, isovalerate, valerate
		Euryarchaeota	↓ *Methanosphaera*	(+) isobutyrate, isovalerate, valerate
			↓ *Methanobrevibacter*	(+) isovalerate, valerate
		Bacteroidota	↑ *Prevotella*	(+) butyrate
		Proteobacteria	↑ *Escherichia-Shigella*	(−) acetate

↑ indicates an increase in value. ↓ indicates a decrease in value. (+) indicates statistical significance positive correlation at *p* < 0.05. (−) indicates statistical significance negative correlation at *p* < 0.05. Abbreviation: PS, protein substitute group; WE, whole egg group.

## Data Availability

Data are contained within the article and Supplementary Materials.

## Supplementary Materials

The following supporting information can be downloaded at: https://www.mdpi.com/article/10.3390/nu15224804/s1, Figure S1: Impact of Dietary Intervention on Individual SCFA Levels. Box plots represent mean change (difference week 35 − week 0) of SCFAs (μmol/g). (A) Mean change of control group (B) Mean change of protein substitute group (C) Mean change of whole egg group; Figure S2: Heatmap of Pearson’s coefficient between top 42 differential abundance of gut microbiota and concentrations of SCFAs in all group. The bacterial genus is sorted from negative (brick red squares) to insignificant (white squares) to positive (brick green squares) correlation. Significant correlations are marked by * (correlation coefficient ≥ ±0.3). Abbreviations: (A) = Actinobacteriota; (F) = Firmicutes; (C) = Cyanobacteria; (P) = Proteobacteria; (E) = Euryarchaeota; (B) = Bacteroidota.
