# Supplementary material for: Influence of Prolonged Whole Egg Supplementation on Insulin-like Growth Factor 1 and Short-Chain Fatty Acids Product: Implications for Human Health and Gut Microbiota
Source: Nutrients. 2023 Nov 16;15(22):4804. doi: 10.3390/nu15224804 (PMC10674712; doi:10.3390/nu15224804)
Supplement: Supplementary file 1 [file nutrients-15-04804-s001.zip › nutrients-2686924-supplementary.pdf]

Supplement Method S1: Bacterial DNA extracted from stool sample

The QIAamp® PowerFecal® Pro DNA Kit (QIAGEN, Hilden, Germany) was used (following to the manufacturer). In a PowerBead Pro Tube, 250 mg of stool was weighed, and 800 µL of solution CD1. Vortex using PowerLyzer 24 Homogenizer at maximum speed for 10 minutes. and centrifuged at 15000 × g for 1 minute. Pipette 600 µL of the supernatant into a microcentrifuge tube and 200 µL of solution CD2 was added after centrifugation. Transfer 700 µL of supernatant to a microcentrifuge tube and add 600 µL of solution CD3. The lysate (650 µL) was loaded into the MB spin column and centrifuged. The flow-through was discarded, the MB spin column was placed into a collection tube, and 500 µL of EA solution was added. The flow-through was then centrifuged and discarded. Then, 500 µL of solution C5 was added to the MB spin column, centrifuged, and the flow-through discarded. The MB spin column was carefully centrifuged for 2 minutes at 16000 × g. Finally, 50 µL of solution C6 and centrifuge at 15000 × g for 1 minute. The MB spin column was discarded. DNA is currently ready for downstream applications. Microbial DNA concentration and a purity ratio (acceptable in range 1.8 – 1.9) were measured using FLUOstar Omega (software version 5.5 R4, BMG LABTECH, Ortenberg, Germany).

The samples were sent to the Centre d'expertise et de Services Génome Québec (Génome Québec, Montréal, Canada) for 16S rRNA sequencing. The gut microbiome study used the NovaSeq 6000 platform and Illumina sequencing by synthesis (SBS) to generate low error rate amplicon data. The analysis focused on V4 of the 16s rRNA genes, where 515F – 806R was used as a primer. AmpliconSeq sequencing was performed on the NovaSeq platform (Génome Québec, Montréal, Canada). The raw sequence reads were processed using QIIME2 version 2021.4 and operated according to the standard pipeline recommended by Hall and Beiko. Briefly, the primers were trimmed and sequences with a quality score lower than 30 were filtered and further analyzed by merging the forward and reverse reads. The chimera was defined and removed by testing at least six abundances as potential parents. Taxonomy was classified with at least 97% sequence similarity to the Silva v132 database. The amplicon sequence variant (ASV) with an abundance of less than two sequences was filtered out. Phylogenetic analysis was performed by aligning the ASVs using MAFFT and a tree with FastTree. Finally, alpha and beta diversity and differential abundance were analyzed and visualized using the R package. We generated a principal coordinate analysis (PCoA) using the weighted UniFrac distances between the baseline (T1) and the endpoint (T3) of each group. The analysis of the composition of microbiomes with bias correction (ANCOM-BC) was applied to analyze the differential abundance in this study. We used pairwise comparisons with false discovery rate (FDR) correction to estimate the differences in taxonomic composition between the time points of each host group. The ASVs classified by taxonomy from the previous step were imported into the R platform. We used the observed ASVs, Chao1 richness, Shannon index, and the InvSimpson index for the alpha diversity estimator. These were used to characterize the diversity of species among groups. PCoA was generated from weighted UniFrac distances for the beta diversity analysis.

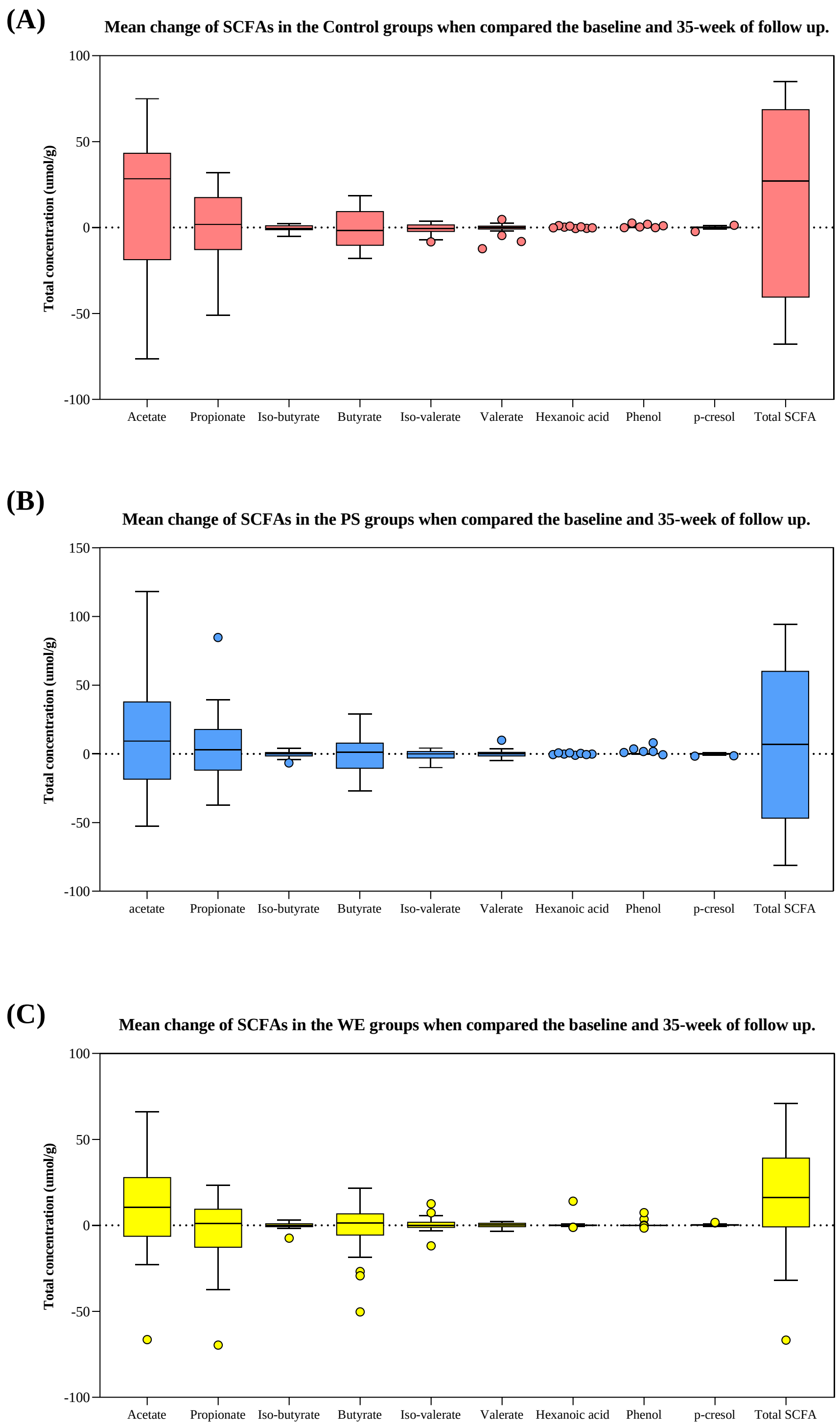

Supplement Figure S1. Impact of Dietary Intervention on Individual SCFA Levels. Box plots represent mean change (difference week 35 – week 0) of SCFAs (µmol/g). **(A)** Mean change of control group **(B)** Mean change of protein substitute group **(C)** Mean change of whole egg group.

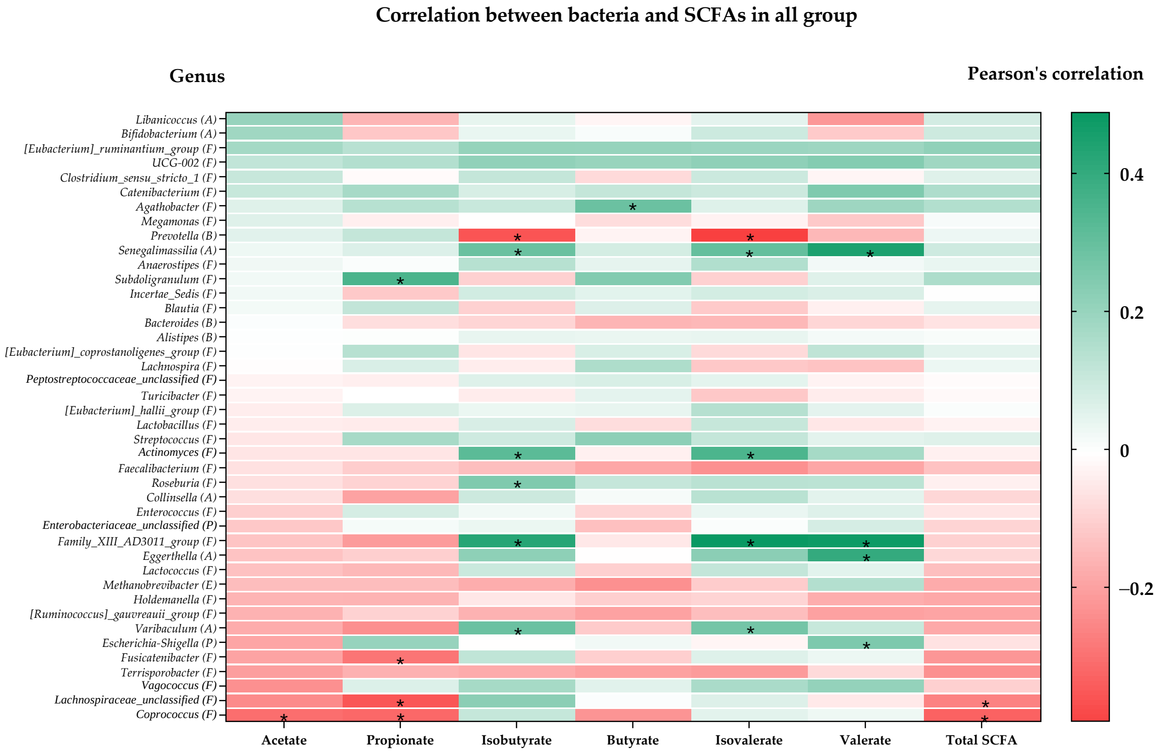

Supplement Figure S2. Heatmap of Pearson's coefficient between top 42 differential abundance of gut microbiota and concentrations of SCFAs in all group. The bacterial genus is sorted from negative (brick red squares) to insignificant (white squares) to positive (brick green squares) correlation. Significant correlations are marked by *(correlation coefficient ≥ ±0.3). Abbreviations: (A)=Actinobacteriota; (F)=Firmicutes; (C)=Cyanobacteria; (P)=Proteobacteria; (E)=Euryarchaeota; (B)=Bacteroidota.
